# Do Inflammations Require a Different Treatment from That Pursued Fifteen or Twenty Years Ago?

**Published:** 1859-02

**Authors:** B. S. Woodworth

**Affiliations:** Fort Wayne, Ind.


ARTICLE II.

### HAS THERE BEEN A "CHANGE OF TYPE" IN INFLAMMATORY DISEASES WITHIN THE LAST TWENTY YEARS;

OR, IN OTHER WORDS,

### "DO INFLAMMATIONS *REQUIRE* A DIFFERENT TREATMENT FROM THAT PURSUED FIFTEEN OR TWENTY YEARS AGO?"

BY B. S. WOODWORTH, M.D., FORT WAYNE, IND.

The above question has been very extensively discussed within a year past by many eminent physicians, and especially in Edinburgh, by Dr. Allison on the one side, and Prof. Bennett on the other. Many arguments have been produced by both parties in support of their respective doctrines, but I have myself no hesitation in saying that Prof. Bennet has the best of the argument. That *mankind* has changed its type, in the course of thousands of years, may be possible, although here we find two schools of anthropology, one maintaining that it has so changed, and the other that it has not. Thus, Mr. Whitehead, in his book "On Transmission from Parent to Offspring of some forms of Disease, and of Morbid Taints and Tendencies," says: "It is by no means unreasonable to suppose that races, now the most widely dissimilar, may owe their distinguishing characteristics to time and circumstances (external environments), and that as the divergence has been constantly widening, so their approximation may be supposed to have been much nearer in ages gone by. We certainly have no records of that antique date of that high contrast between races which is now known to exist between the African and the European." But, on the other hand, the other school maintain, "that so far back as history goes, the species of animals, as we call them, have not changed. The races of men have been absolutely the same—they were distinct then for that period as at present." But admitting that mankind has not changed its type, has it even *degenerated* within the last two or three thousand years?—then an advocate for, and against this, the progressive degeneracy of the human race.

But that diseases have so materially changed their type within the last twenty years as to require a treatment diametrically opposite to that required then, seems to me so manifestly absurd as to hardly admit of an argument to controvert it.

That the treatment of inflammation and fevers, by all enlightened physicians, has undergone a great change within a few years, is well known; but, while one party attributes the result to a change in the character of these diseases—a change of type, as it is called,—the other party attributes the change to the diffusion of more correct views of pathology and improved powers of diagnosis, as well as from witnessing the better effects of the less heroic non-anti-phlogistic and less perturbative treatment now employed. The profession are, doubtless, somewhat indebted to the various medical delusions, especially that miserable delusion, homœopathy, for the knowledge of what nature can accomplish when aided by a proper regiminal course. And we are also somewhat indebted to the hydropathist for showing us (what many knew before, for, as Charles Lamb said, "it was as old as the deluge, and," he added, "like it, it killed more than it cured,") that water is also a valuable curative agent when judiciously used.

The propositions laid down by Professor J. Hughes Bennett seem to me so perfectly clear that I will transcribe them, as some of your readers may not have seen them.

*Proposition* 1*st.* That little reliance can be placed on the experience of those who, like Cullen and Gregory, were unacquainted with the nature of, and the mode of, detecting internal inflammations.

*Prop.* 2*d.* That inflammation is the same now as it has ever been, and that the analogy sought to be established between it and the various types of fevers is fallacious.

*Prop.* 3*d.* That the principles on which bloodletting and anti-phlogistic remedies have hitherto been practised are opposed to a sound pathology.

*Prop.* 4*th.* That an inflammation once established cannot be cut short, and that the only end of judicious medical practice is to conduct it to a favorable termination.

*Prop.* 5*th.* That all positive knowledge of the experience of the past, as well as the more exact observations of the present day, alike establish the truth of the preceding principles as guides for the future.

It is but just to say that Drs. Allison, Gardiner, Watson and others, attempt to controvert these doctrines, but, as I think, with but little success.

Inflammation has been truly represented to be "a twofold process of increased decomposition and increased formation, having its source in a diminution of the vital control over the coerced chemical affinities in the living textures,"—or, in other words, "an altered nutrition." There is in inflammation a general lowering of the vital powers; and this being so, how absurd must be that practice which attempts to cure it by reducing the vital powers still lower, and thus retarding, if not preventing, the efforts of nature towards a cure.

I confess that I have of late years become somewhat tinctured with the ideas of Sir John Forbes, Dr. J. Bigelow and others, who believe that it is not best to meddle much with nature in *any* disease, at least, "till she indicates the proper course to pursue."

Twenty years ago, when I first settled in the Maumee Valley, it was the fashion to treat all inflammatory diseases on the strictest anti-phlogistic, most heroic way. Bleeding, tart. emetic and mercury, were the sheet-anchors in almost all cases; nor was this treatment confined to purely inflammatory diseases. It was the custom to bleed at the commencement of most of the cases of remittent fever, as well as some of the intermittents. Most practitioners there thought that remittents required a materially different plan of treatment from intermittents; and they treated them in the Eberle manner, with bleeding, calomel, nitrate potash, ipecac, etc., *ad nauseum*, with the result of seeing the original fever, after a few days, changed into a mercurial fever which lasted for an indefinite length of time, and which generally ran into a chronic diarrhœa. If the patient happened to have sufficient recuperative energy, he recovered only after a very tedious convalescence from a disease mainly produced by the medicines. Now, under the improved treatment by quinine, and, perhaps, some mild cathartics, this same class of cases is convalescent in a couple of days, and well in a week. I need not say that the physician who then would have had the presumption to treat a case of pneumonia, peritonitis, or any other acute inflammation, without bleeding or mercury, would have been considered guilty of the grossest mal-practice, and been denounced by his professional brethren for his temerity. Although the more *sanguinary* part of this treatment has been almost universally abandoned, I am sorry to say that the treatment by mercurials and drastic cathartics is still too much in vogue in this section of the State of Indiana, especially in the rural districts. This is owing to a want of light on the subject—to an ignorance of what is going on in the medical world. The so-called regular physicians, at least, many of them, do not take the medical journals, nor buy new medical books, and thus keep up with the progressive knowledge of pathology, therapeutics, etc., etc.

Erysipelas is a disease which we used to treat in an antiphlogistic way—a disease which is as truly one of debility as the lowest form of typhus or typhoid fever. Under the antiphlogistic treatment many cases of this disease died; whereas, scarcely one per cent of sporadic cases would die if left to the unassisted efforts of nature, and not so many by a judicious tonic and stimulating plan. By way of parenthesis, I would say that I think that there is too much importance given to the local treatment of this disease, and a great deal has been written about it. Tr. iodine, nitrate silver, sulph. iron, collodion, etc., etc., each have their advocates, who seem to think that they are of more importance than the constitutional remedies. As it is a constitutional and self-limited disease, I never could conceive that local applications were of much more use than they would be in small pox; and I never could see any other use of tr. iodine and nit. silver but to make the patient look horribly, and impress the vulgar with the terrible nature of the disease.

As to whether inflammations have changed their types in the Maumee and Wabash Valleys within twenty years, I should say, from my observations during the above mentioned period, that if there be any difference they have assumed a more sthenic rather than asthenic character. This will readily be believed by any physician when he is told that intermittent fever, and malarious diseases generally, once so universal here, are now not nearly so common.

Enlargement of the spleen, and its resulting (or accompanying) anemia, dropsies, etc., are now comparatively rare—in short, that typhoid symptoms, in most diseases, supervene at not so early a period as formerly. Perhaps this may be owing partly to the improvement in the treatment; still there can be no doubt that in this part of the State the inhabitants generally are more robust and vigorous than they were twenty years ago. And probably the same may be said of various other parts of the United States, which were once so unhealthy that no one lived to be over twenty-seven years of age, as I read in some old medical book concerning Petersburg in Virginia.

There are now, doubtless, some debilitating and degenerating causes in operation to counterbalance, in some degree, the absence of the malarious influence. The greatest of all these causes is the use of intoxicating drinks, which has been fearfully on the increase for a few years past. Who now does not drink, at least, lager bier? What gentleman (?) that travels does not carry his bottle of brandy? And then what immense numbers of men (and women too) are addicted to the use of opium, tobacco, etc. If these stimulants and narcotics could only be banished from the face of the earth, and venereal diseases be treated in a more rational manner (without mercury), I think there might be some hope that mankind might be partially regenerated (physically). I do not suppose that all the unhallowed passions of men would be banished if intoxicating drinks were disused, but the greatest of all of the excitants of these passions would be withdrawn. That a great change in the general health of its inhabitants for the better has taken place in the Wabash and Maumee Valleys within twenty years is perfectly obvious. A person visiting the towns in these valleys some years ago, would have seen the exact counterparts of Eden (described by Dickens in Martin Chuzzlewit) in almost all of them. I well recollect many of these Edens, with their paradisical environments and their happy (?) inhabitants.

The usual treatment by the physicians here in pneumonia, up to the time I came here to reside, was, as I said before, by bleeding, tart. emetic, calomel, blistering, etc. The year before I came here this disease—pneumonia—was epidemic, and *all* the first cases, some thirty in number, died, which, of course, created quite a panic. These were treated in the strictest anti-phlogistic way. An opposite course of treatment was then instituted by some practitioners, who had the good sense to see that a course of treatment which resulted so unfavorably must be wrong. By the change in the treatment the patients recovered, and the disease was stripped of its terrors. Since this period, the lancet has been but seldom used here, but a more rational treatment has been pursued.

I know that the epidemic constitution of diseases, as it is called, differs in different epidemics; but I think it would be difficult to show that in any epidemic of an inflammatory or any other disease, an anti-phlogistic treatment would be proper.

When I look back to the old treatment of inflammations (as well as fevers), I am horror struck, and wonder at the prejudice which could have continued so barbarous a practice so long.

And now, to all the old fogies of the blood-letting, mercuralizing, drastic catharticising, blistering, burning, cauterizing, and (universally) overdrugging school, I would recommend to read Sir John Forbes' book "On Nature and Art in the Cure of Disease;" Dr. Bigelow's little book lately published; Professor Bennett's late work, "Clinical Lectures on Medicine,"—and see if these will not somewhat dilute and modify their barbarous practice. And it is to be hoped that we shall all—if we have not already—discard from our minds the preconceived notions that we imbibed from reading the old standard treatises on practice; and then I think that we may have the consolation to think, that if our patients do die, that they, at least, will die *natural* deaths.

